# A Bioclimate-Based Maximum Entropy Model for *Comperiella calauanica* Barrion, Almarinez and Amalin (Hymenoptera: Encyrtidae) in the Philippines

**DOI:** 10.3390/insects12010026

**Published:** 2021-01-04

**Authors:** Billy Joel M. Almarinez, Mary Jane A. Fadri, Richard Lasina, Mary Angelique A. Tavera, Thaddeus M. Carvajal, Kozo Watanabe, Jesusa C. Legaspi, Divina M. Amalin

**Affiliations:** 1 Biology Department, College of Science, De La Salle University, Taft Avenue, Manila 1004, Philippines; mary_angelique_a_tavera@dlsu.edu.ph; 2 Biological Control Research Unit, Center for Natural Science and Environmental Research, De La Salle University, Taft Avenue, Manila 1004, Philippines; 3 Biology Department, College of Arts and Sciences, Romblon State University, Odiongan, Romblon 5505, Philippines; maryjanefadri@gmail.com; 4 Philippine Coconut Authority-Zamboanga Research Center, San Ramon, Zamboanga 7000, Philippines; richard.lasina.research@gmail.com; 5 Center for Marine Environmental Studies, Ehime University, Matsuyama, Ehime 790-8577, Japan; tads.carvajal@gmail.com (T.M.C.); watanabe.kozo.mj@ehime-u.ac.jp (K.W.); 6 Center for Medical, Agricultural and Veterinary Entomology, United States Department of Agriculture-Agricultural Research Service, Tallahassee, FL 32308, USA; jesusa.legaspi@ars.usda.gov

**Keywords:** *Aspidiotus rigidus*, *Comperiella calauanica*, Maxent, pest invasion forecasting, species distribution modeling

## Abstract

**Simple Summary:**

The discovery of *Comperiella calauanica* a parasitoid confirmed to be the major natural enemy of the invasive diaspidid, *Aspidiotus rigidus*, has led to the promise of biological control in sustainable pest management of this devastating coconut pest. In this study, we employed Maximum Entropy (Maxent) to develop a bioclimate-based species distribution model (SDM) for the parasitoid from presence-only data recorded from field surveys conducted in select points the Philippines. Results of assessment of the generated model point to its excellent power in predicting either suitability of habitat, or potential occurrence or distribution of *C. calauanica*. Since the parasitoid is highly host-specific, the model may also apply to *A. rigidus*. Field surveys in select areas in the Philippines confirmed the occurrence of the invasive coconut scale in areas predicted by the model as having considerable probability of occurrence, or habitat suitability. Our findings strongly suggest the potential utility of Maxent SDMs as tools for pest invasion forecasting and GIS-aided surveillance for integrated pest management (IPM).

**Abstract:**

*Comperiella calauanica* is a host-specific endoparasitoid and effective biological control agent of the diaspidid *Aspidiotus rigidus*, whose outbreak from 2010 to 2015 severely threatened the coconut industry in the Philippines. Using the maximum entropy (Maxent) algorithm, we developed a species distribution model (SDM) for *C. calauanica* based on 19 bioclimatic variables, using occurrence data obtained mostly from field surveys conducted in A. rigidus-infested areas in Luzon Island from 2014 to 2016. The calculated the area under the ROC curve (AUC) values for the model were very high (0.966, standard deviation = 0.005), indicating the model’s high predictive power. Precipitation seasonality was found to have the highest relative contribution to model development. Response curves produced by Maxent suggested the positive influence of mean temperature of the driest quarter, and negative influence of precipitation of the driest and coldest quarters on habitat suitability. Given that *C. calauanica* has been found to always occur with *A. rigidus* in Luzon Island due to high host-specificity, the SDM for the parasitoid may also be considered and used as a predictive model for its host. This was confirmed through field surveys conducted between late 2016 and early 2018, which found and confirmed the occurrence of *A. rigidus* in three areas predicted by the SDM to have moderate to high habitat suitability or probability of occurrence of *C. calauanica*: Zamboanga City in Mindanao; Isabela City in Basilan Island; and Tablas Island in Romblon. This validation in the field demonstrated the utility of the bioclimate-based SDM for *C. calauanica* in predicting habitat suitability or probability of occurrence of *A. rigidus* in the Philippines.

## 1. Introduction

The Philippines is a primarily agricultural nation in Southeast Asia, despite rapid industrialization in many areas of the archipelago. Statistics in 2015 indicate that 29.15% of total employment in the Philippines is in agriculture [1]. The agricultural sector has provided the fourth highest contribution to the country’s gross domestic product (GDP), with the latest data summarized by the Philippine National Statistics Coordination Board indicating GDP from agriculture at 53.7 billion Philippine pesos (equivalent to about 1 billion US dollars). Coconut is one of the high value commercial crops of the country and has been recognized for years as a top agricultural export [2]. However, production of this crop was severely threatened by an outbreak of the destructive coconut scale, *Aspidiotus rigidus* Reyne (Hemiptera: Diaspididae), which devastated plantations in the Southern Tagalog region of Luzon Island from 2010 to 2015. Feeding of this diaspidid on the foliage of coconut palms has been found to impair photosynthesis, consequently affecting flowering, fruiting, and even compromising the survival of the infested tree [3].

A native parasitic wasp belonging to genus *Comperiella* Howard (Hymenoptera: Encyrtidae) was discovered and subsequently found to effectively parasitize *A. rigidus* in the outbreak areas from 2014 onwards. Preliminary findings and observations from field and laboratory studies suggested the potential of the parasitoid for biological control [4]. Additionally, the encyrtid was not only the first native record in the Philippines for its genus, but was also described as a new species, *C. calauanica* Barrion, Almarinez and Amalin [5]. *C. calauanica* has been found to be very specific to *A. rigidus*, although mathematical modeling and simulations by Palen et al. [6] assumed that the parasitoid may exhibit a Holling type III functional response in which parasitism on an alternate host is necessary for survival in the absence of the primary host. Management of *A. rigidus* outbreaks in the Southern Tagalog region of Luzon Island as well as in Zamboanga Peninsula in Mindanao has been reportedly a result of biological control by *C. calauanica*, owing to its high host-specificity and putatively host density-dependent parasitism in the field [7].

Published information on the distribution of *C. calauanica* and *A. rigidus* in the Philippines has been limited, and has so far been based either on field studies conducted in *A. rigidus* outbreak areas [4,5], or on reports by local experts and agencies [3]. Distribution modeling to identify or predict suitable habitats outside known outbreak areas has yet to be explored for these two insect species. Predictive geographical modeling that is based on the dependence of species and community distributions on environmental factors has been viewed as an important means to assess the impact of natural and anthropogenic environmental change on the distribution of organisms [8]. In addition, climate-based ecological models can help in conservation efforts by providing information for resource and habitat management [9]. Recently, distribution modeling has been used to predict areas of high risk brought about by human infrastructures [10], as well as in the identification of potential niche areas for habitation of species that may have an ecological service of medical importance to humans [11]. Potential distributions of invasive species can also be predicted with the aid of species distribution models (SDMs) [12,13]. Among the popular algorithms used in modeling species distributions is the maximum entropy (Maxent) approach, which requires presence-only data as an indication of the species’ occurrence. Models produced using Maxent can be easily understood and interpreted, and provide valuable insights into distribution and habitat suitability for a species [14,15], including under future conditions possibly impacted by climate change [16].

Insect population distributions are largely affected by abiotic conditions in the environment, including climate. Particular species of insects have their own characteristic tolerance to climatic factors, and changes in such factors can lead to potential changes in distribution [17]. In view of this, Maxent modeling has been used to predict the current and potential distributions of invasive species [18], as well as those of a variety of forest and agricultural insect pests which include: the large pine weevil, *Hylobius abietis* L., and the horse-chestnut leaf miner, *Cameraria ohridella* Deschka and Dimič [19], and six tephritid fruit flies [20] in Europe; three species of tephritid flies under genus *Dacus* Fabricius [21] and the European grapevine moth, *Lobesia botrana* Denis and Schiffermüller, in China [22]; the ricaniid planthopper, *Ricania shantungensis* Chou and Lu in Korea [23,24]; the cotton mealybug, *Phenacoccus solenopsis* Tinsley, in India [25] and worldwide [26]; and the invasive European paper wasp, *Polistes dominula* Christ in the southern hemisphere [13].

The use of Maxent modeling as a tool in integrated pest management, particularly in forecasting potential areas of new pest invasion relative to climate, has not yet been explored very well in the Philippines, or in Southeast Asia. Hence, in view of the use of *C. calauanica* for biological control of *A. rigidus*, the Maxent approach was employed in this study to generate a bioclimate-based SDM for the prediction of either the presence of the parasitoid or suitability of areas for its occurrence. This study provides a window into the potential of bioclimate-based SDMs as tools for integrated pest management, especially in view of climate change. The ability and utility of the distribution model of a highly specific parasitoid to predict the potential distribution or areas of new invasion by its host are likewise demonstrated.

## 2. Materials and Methods

### 2.1. Species Presence, Bioclimatic Variables, and Other Data

Presence-only data pertaining to occurrence of *C. calauanica* were derived from GPS coordinates recorded from periodic field surveys conducted from April 2014 to June 2016 in 15 sampling points across three provinces (Batangas, Cavite, and Laguna) in the Southern Tagalog region, and in 4 points in the town of Orani in Bataan in the Central region of Luzon Island (Table 1). An additional coordinate was derived using Google Maps (accessible from http://maps.google.com) to represent a point in Isabela City, Basilan Island where sightings of *C. calauanica* were reported in January 2016 but were not actually covered by our surveys. The occurrence points were encoded in spreadsheet form (with three columns for species, longitude, and latitude in that order) using Microsoft Excel and saved as a comma-separated values (CSV) file. A second set of presence-only data containing 13 occurrence points recorded from subsequent field surveys conducted in Zamboanga City in April and August 2017 was encoded into another CSV file to provide the points for model testing. 

Bioclimatic data sets were downloaded from the WorldClim Global Climate Database [27]. These bioclimatic data were derived from global climate data interpolated by Hijmans et al. [28] and represent current conditions. The downloaded raster data sets, in BIL format with 30 arc-seconds resolution, pertain to 19 variables (Table 2). For visualization of the SDM and subsequent map construction, vector layers (in SHP format) of the administrative boundaries of the Philippines were directly downloaded from the Philippine GIS Data Clearinghouse [29].

### 2.2. Maxent Species Distribution Modeling for C. calauanica

Maxent Version 3.3.3k was used to develop the SDM for *C. calauanica*. The presence-only data encoded in CSV served as the sample, while the downloaded bioclimatic data sets in BIL format were used as the environmental layers for model construction. Among the 20 presence records inputted into the algorithm, 18 were used for model training. Iterations of the optimization algorithm were set to 5000, and Jackknife test was included in the algorithm to provide a measurement of the importance of each bioclimatic variable in the model. Model testing was performed using the second set of 13 occurrence points from 2017. Response curves were also generated for assessment of the variables. Two runs of the same model were done so that the first output was set to express values logistically and the second with values set to raw. The outputs in raster form (in ASC format) were visualized, enhanced, and assessed in combination with other geospatial datasets through Quantum GIS (QGIS) Versions 1.8.0 and 3.6.0.

### 2.3. Analysis and Assessment of the Species Distribution Model

The constructed Maxent model was evaluated using the result of the receiver operating characteristic (ROC) analysis, with the obtained values for the area under the ROC curve (AUC) serving as a measure of model performance. AUC values closer to 1.0 indicate better model performance compared to those further from 1.0. The testing AUC is considered as the true indicator of the predictive power of the model [30]. The bioclimatic variable with the highest percentage contribution to the construction of the model was likewise noted. To infer which among the bioclimatic variables the species appear to respond to most positively (i.e., preferred conditions) and to which they respond most negatively (i.e., conditions to which they appear to be most sensitive) in terms of their occurrence, the trends shown in the response curves of a model were examined and compared with each other. Variables whose response curves showed a clearly unidirectional upward or downward trend were considered to be those with putatively greatest impact on potential distribution.

### 2.4. Validation of the Predicted Distribution of A. rigidus

A field survey for *A. rigidus* surveillance was conducted in Zamboanga City and in Isabela City in Basilan Island in Western Mindanao initially from November 2016. Subsequent surveys were done in late January to early February 2017 for field release of mass-reared *C. calauanica*, and in April 2017 for monitoring of establishment and spread of the parasitoids. Samples of coconut fronds were randomly collected using the Rapid Ground Assessment (RGA) method developed by the Philippine Coconut Authority (2018, unpublished). In this method, two to five trees were randomly selected in selected areas in Zamboanga City and Isabela City with reports of *A. rigidus* infestation, and where mass-reared *C. calauanica* were released as biological control agent [7]. Field surveillance was also conducted in Tablas Island in the province of Romblon in January 2018 for confirmation of received verbal reports of *A. rigidus* infestation. Presence of *A. rigidus* or *C. calauanica* was confirmed by in situ inspection of samples for colonies of *A. rigidus* or occurrence of adult and immature stages of *C. calauanica* among the scale colonies, or by ex situ inspection of unparasitized and parasitized scale colonies present on laboratory-processed samples or high resolution scanned images of leaflet samples [7]. GPS coordinates of all of the points where *A. rigidus* infestations were confirmed were recorded and encoded in a CSV file for overlaying of these points on the map with the *C. calauanica* SDM in QGIS. Incidence of points on areas predicted by the SDM to have non-zero probability (or at least low-moderate suitability) was considered validation of the prediction of occurrence.

## 3. Results

### 3.1. Maxent Species Distribution Model for C. calauanica

**Figure 1:**
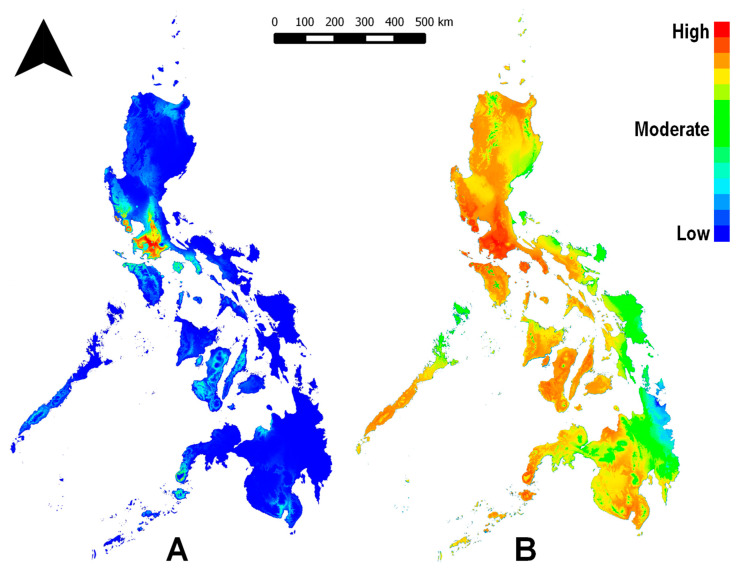
Bioclimate-based species distribution model (SDM) for *Comperiella calauanica* in logistic (**A**) and raw (**B**) expressions of calculated probabilities. Warmth of color indicates relative probability of occurrence or suitability of habitat.

The generated bioclimate-based distribution model for *C. calauanica* (Figure 1) predicts hotspot areas in the provinces of Southern Luzon where the outbreak of *A. rigidus* between 2010 and 2015 most heavily devastated coconut plantations and stands: Batangas, Cavite, and Laguna. It additionally predicts hotspots in the province of Bataan in Central Luzon. These predicted hotspots were expected since all of the survey points in the study, which were inputted into the modeling algorithm, were in those provinces. Areas with non-zero habitat suitability were nonetheless predicted in other parts of the Philippine archipelago that were outside the range of the survey points. Although the model in raw expression (Figure 1B) shows predictions of moderate to high habitat suitability throughout almost the entire archipelago, areas whose predicted probabilities may be considered substantial (between “low-moderate” and “high”) consistent with the logistic expression (Figure 1A) include: several other parts of Luzon mainland; other islands in the Luzon island group, notably Mindoro, Palawan, Marinduque, Romblon, and Masbate; Panay Island; Negros Island; Cebu; Bohol; several parts of Mindanao mainland, particularly the southwestern Zamboanga Peninsula; Basilan Island; and Sulu. 

### 3.2. Analysis and Assessment of the Species Distribution Model

The training AUC value of the *C. calauanica* SDM was 0.996, and the test AUC value was 0.966 (standard deviation = 0.005). The *C. calauanica* SDM, therefore, has very high predictive power based on these AUC values. Since *C. calauanica* has been found to be very specific to its host and has been found to occur where its host is, its SDM may also have considerable ability to predict the distribution of (or habitat suitability for) *A. rigidus*.

**Figure 2:**
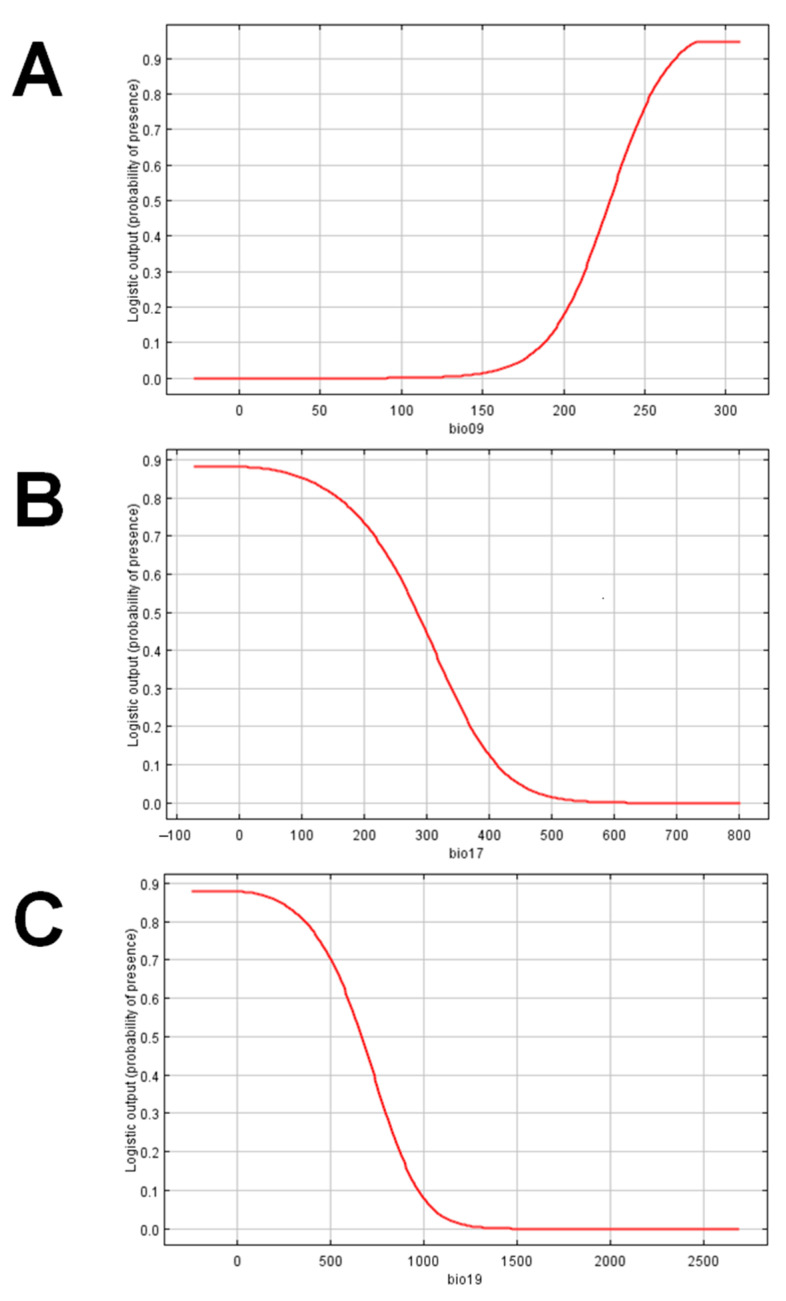
Response (probability of presence) of *Comperiella calauanica* to selected bioclimatic variables: (**A**) mean temperature of the driest quarter; (**B**) precipitation of the driest quarter; and (**C**) precipitation of the coldest quarter.

Precipitation seasonality (bio15) was the bioclimatic variable found to have the highest relative contribution in the development of the model at 51.5%. Three variables, namely mean temperature of the driest quarter (bio09), precipitation of the driest quarter (bio17), and precipitation of the coldest quarter (bio19), were found to have clear unidirectional upward or downward trends, and therefore potentially have the greatest impact on occurrence (Figure 2).

### 3.3. Validation of the Predicted Distribution of A. rigidus

**Figure 3:**
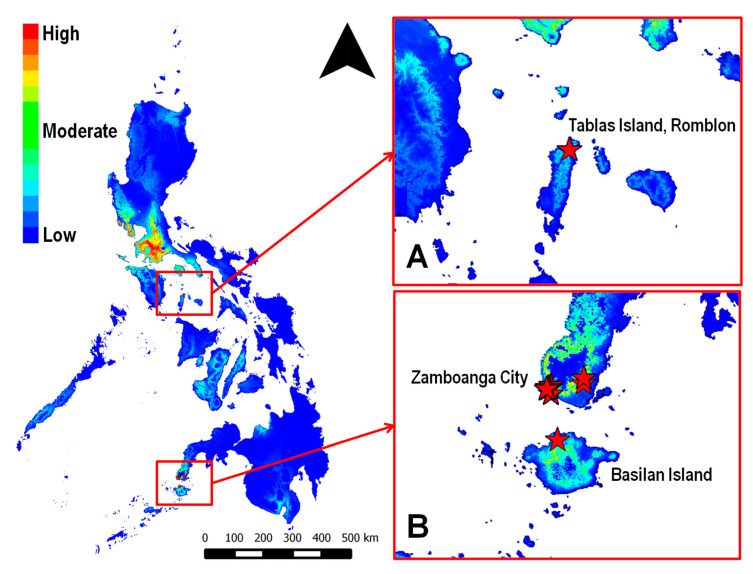
Maxent SDM of *Comperiella calauanica*, with areas in Romblon (**A**) and Western Mindanao (**B**) where infestations of *Aspidiotus rigidus* had been confirmed. Red stars mark the points where confirmatory surveys were conducted between November 2016 and January 2017.

**Figure 4:**
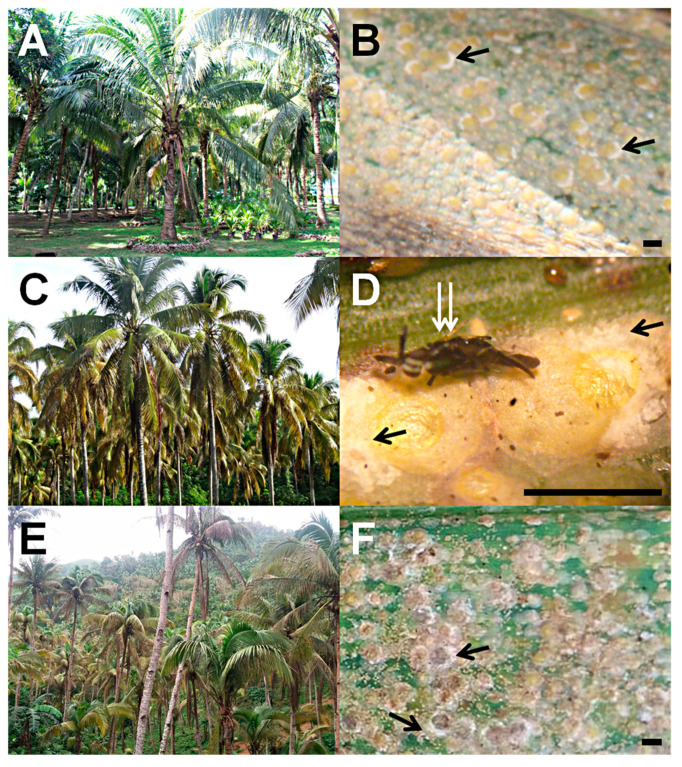
*Aspidiotus rigidus*-infested coconut trees with magnified view of scale colony samples from: Zamboanga City (**A**,**B**); Isabela City, Basilan (**C**,**D**); and Tablas Island, Romblon (**E**,**F**). Black arrows point to the characteristic distribution of eggs and egg skins along the pygidial ends of mature female *A. rigidus* which can be used as basis to quickly distinguish this species from other *Aspidiotus* spp. on coconut. White double arrow points to female *Comperiella calauanica*. Scale bars on the photomicrographs approximate 1.0 mm.

*In situ* and ex situ examination of coconut leaflet samples collected during field surveillance confirmed the occurrence of *A. rigidus* in areas outside mainland Luzon, namely Zamboanga City, Isabela City in Basilan Island, and Tablas Island, Romblon (Figure 3). *A. rigidus* without *C. calauanica* was confirmed in Zamboanga City (Figure 4A,B) and in Tablas Island (Figure 4E,F), whereas *C. calauanica* was confirmed to parasitize *A. rigidus* in Isabela City (Figure 4C,D). These points with confirmed occurrence coincided with areas predicted with moderate to high habitat suitability by the SDM. The infestation in Zamboanga City at the time of the initial surveillance was not yet at the outbreak level, whereas an outbreak appeared to have already started in Tablas Island by the time surveillance under this study was carried out.

## 4. Discussion

The coconut scale, *A. rigidus*, has emerged as a serious, invasive pest of coconut in various parts of the Philippines. A few years after the diaspidid was first reported in the country as the species which caused an outbreak in 2010 [3], the native encyrtid *C. calauanica* was found to be a candidate biological control agent for pest management. We developed a Maxent SDM primarily to predict habitat suitability for *C. calauanica* in case it were released in the field for management of its host. The encyrtid has been found to be very specific only to *A. rigidus*, although mathematical modeling with simulations assumed Holling type III functional response [6] which would require *C. calauanica* to parasitize an alternate host in the absence of *A. rigidus.* An alternate host has not been found so far, and the parasitoid is so far known to parasitize only *A. rigidus* [7]. Hence, it is reasonable to view the SDM for *C. calauanica* as a predictive model that may also apply to its primary host, especially in view of the confirmed occurrence of *A. rigidus* in all of the encyrtid’s 20 occurrence points that served as input to the modeling algorithm.

The model’s training and test AUC values of 0.996 and 0.966, respectively, are higher than 0.8, the value above which the AUC must be in order for the predictive ability of the model to be considered “convincing” [31]. If the model is viewed as a habitat suitability model for *A. rigidus*, the predicted areas with non-zero probabilities of occurrence could therefore be considered as potential risk areas for new invasion. This is most especially true for the peninsula of Zamboanga. Since *A. rigidus* is naturally wind-dispersed [3], the likelihood of invasion by the pest coming from the nearby island of Basilan is very high.

We presented in Figure 1 the SDM both in logistic and raw expressions of probabilities. It should be noted that raw values tend to be significantly lower than their logistic equivalent. Given the spectral scale for qualitative interpretation of colors on the SDM, the predicted probability value for a given point could be considered “high” when expressed as raw, but only “moderate” when logistically expressed. This would explain the apparent spectral discrepancy between the raw and logistic expressions of the same SDM.

Our finding of precipitation seasonality (bio15) as having the highest relative contribution in model development suggests that distribution of *C. calauanica* or *A. rigidus* may be influenced more by precipitation than by temperature, especially considering that the Philippines is a tropical country, where temperatures throughout the year tend to vary less than in temperate regions. In comparison, variables pertaining to temperature or its variations were found to have significant influence on the predicted distributions of insect pest species in temperate regions, namely *Dacus* spp. [21] and *Lobesia botrana* [22] in China, *Ricania shantungensis* in Korea [24], *Hylobius abietis* and *Cameraria ohridella* in Europe [19], and six species of tephritid fruit flies in Europe [20].

The response curves for mean temperature of the driest quarter (bio09), precipitation of the driest quarter (bio 17), and precipitation of the coldest quarter (bio19) (Figure 2) indicate that the probability of occurrence of *C. calauanica* or its primary host increases with higher mean temperatures of the driest quarter, and decrease with higher precipitation during the driest and during the coldest quarters of the year. These findings suggest that the parasitoid or its host could be sensitive to precipitation, and may find habitats with higher mean temperatures and relatively less precipitation to be more suitable. Furthermore, it could be noted in the set of response curves that the predicted probability of occurrence remained constant across changes in variables pertaining to temperature more than precipitation, namely annual mean temperature (bio01), maximum temperature of the warmest month (bio05), minimum temperature of the coldest month (bio06), and mean temperature of the warmest quarter (bio10). If these response curves provide an approximation of the actual ecophysiological responses of either *C. calauanica* or *A. rigidus*, then it is possible that habitat suitability for either insect may be influenced more by precipitation than by temperature.

Presence points used in development of the *C. calauanica* SDM were limited only to the known outbreak and infestation areas from 2014 to early 2016, and were limited to only 20 points, including one that was derived from Google Maps. Findings from field surveys conducted in late 2016 up to 2018 validated these predictions as being, consistent with the high predictive power of the SDM as indicated by the high training and test AUC values computed by Maxent. Previously reported Maxent models for other insect species were developed using between double to a little more than 460 times as many occurrence records (Table 3). Nevertheless, the SDM developed for *C. calauanica* using relatively few points was able to correctly predict the occurrence of *A. rigidus* in Zamboanga City and in Romblon, and together with the parasitoid in Basilan Island. Moreover, infestations of *A. rigidus* were confirmed in the Bicol Region in the southeastern part of Luzon Island [32,33] Maxent has been recognized for being much less sensitive to sample size compared to other distribution modeling algorithms, being able to produce useful, predictive models with as few as 5 occurrence points [34,35]. To date and to our knowledge, this is the first field-based validation of the occurrence or habitat suitability predicted by Maxent SDM for an insect species that is important to agriculture or forestry.

## 5. Conclusions

Maxent was used to develop a bioclimate-based SDM for *C. calauanica*, the highly specific endoparasitoid of the destructive coconut scale, *A. rigidus*, in the Philippines. The SDM predicted moderate to high habitat suitability in areas in Luzon Island as well as in other parts of the archipelago. Some of the hotspots were predicted in areas that were not covered by field surveillance from 2014 to 2016, through which the limited number of occurrence points used in model development was obtained. Despite the relatively small sample size used for model development, the SDM was determined to have an excellent predictive power as indicated by the very high training and test AUC values computed by Maxent. Field surveys conducted from late 2016 to early 2017 confirmed the occurrence of *A. rigidus* in Zamboanga City, as well as in Isabela City in Basilan Island in Western Mindanao, where the SDM predicted hotspot areas. Subsequently, *A. rigidus* was also confirmed through field surveillance in Tablas Island in Romblon, where moderate to high habitat suitability was also predicted. These findings point to the utility of the *C. calauanica* SDM in predicting habitat suitability or probability of occurrence of the coconut pest which caused a devastating outbreak in the Southern Tagalog region of Luzon Island from 2010 to 2015. Bioclimate-based modeling may have a considerable potential as a tool for pest invasion forecasting and GIS-guided pest surveillance. In addition to areas with “high” occurrence probability, those with “low” to “moderate” predicted probability (or habitat suitability) should be treated as potential areas for population establishment, especially if preferable environmental conditions beyond bioclimate (e.g., presence of hosts) may occur in such areas. Modeling based not only on current conditions, but also on projected future conditions should be considered and further assessed. 

Maxent modeling was also able to provide insights into possible responses of *C. calauanica* or *A. rigidus* to climatic factors, particularly precipitation. We recommend that controlled assessments be done to actually determine the ecophysiological responses of either *C. calauanica* or *A. rigidus* to such climatic factors, and verify the Maxent-predicted responses. Sufficient understanding of the ecophysiology of insects, supplemented by valuable information that can be provided by bioclimate-based SDMs, may help in the development of pest invasion risk maps, not only for *A. rigidus* but also for other species of importance to agriculture or forestry in the Philippines and in neighboring Southeast Asian countries, especially in view of climate change.

## Figures and Tables

**Table 1:** Occurrence points of *Comperiella calauanica* recorded from April 2014 to January 2016.

Point	Coordinate (WGS 84)	
	Longitude (°)	Latitude (°)
Batangas, Luzon		
Malvar	121.1466	14.04903
Talisay	121.0107	14.09334
Tanauan	121.0913	14.09887
Sto. Tomas	121.2198	14.05746
Cavite, Luzon		
Silang A	120.9729	14.21884
Silang B	121.0305	14.21385
Tagaytay	121.0002	14.17002
Laguna, Luzon		
Calauan	121.2579	14.09737
Los Baños	121.2595	14.15006
Nagcarlan	121.4137	14.15893
Rizal	121.4109	14.06585
Candelaria	121.4513	13.92844
Alaminos	121.2481	14.06618
San Pablo A	121.2948	14.06757
San Pablo B	121.3333	14.05642
Bataan, Luzon		
Orani A	120.4545	14.76979
Orani B	120.4546	14.76963
Orani C	120.4561	14.77067
Orani D	120.4558	14.77054
Basilan Island, Mindanao		
Isabela City *	121.9947	6.587794

* Interpolated from Google Maps due to inability to be covered by surveys in the current study.

**Table 2:** Bioclimatic variables used in Maxent model development for *Comperiella calauanica* (after Hijmans et al. [23]).

Bioclimatic Variable	Variable Code
Annual mean temperature (°C × 10)	bio01
Mean diurnal range (°C × 10)	bio02
Isothermality	bio03
Temperature seasonality	bio04
Maximum temperature of the warmest month (°C × 10)	bio05
Minimum temperature of the coldest month (°C × 10)	bio06
Temperature annual range (°C × 10)	bio07
Mean temperature of the wettest quarter (°C × 10)	bio08
Mean temperature of the driest quarter (°C × 10)	bio09
Mean temperature of warmest quarter (°C × 10)	bio10
Mean temperature of coldest quarter (°C × 10)	bio11
Annual precipitation (mm)	bio12
Precipitation of the wettest month (mm)	bio13
Precipitation of the driest month (mm)	bio14
Precipitation seasonality	bio15
Precipitation of the wettest quarter (mm)	bio16
Precipitation of the driest quarter (mm)	bio17
Precipitation of the warmest quarter (mm)	bio18
Precipitation of the coldest quarter (mm)	bio19

**Table 3:** Number of occurrence points used for Maxent modeling of selected insect species with respective test AUC values.

Species	Number of Occurrence Points Used in Maxent Modeling	Test AUC	Reference
*Anastrepha fraterculus*	49	0.76 *	Godefroid et al. (2015)
*A. obliqua*	49	0.77 *	Godefroid et al. (2015)
*Bactrocera cucurbitae*	49	0.91 *	Godefroid et al. (2015)
*B. oleae*	49	0.97 *	Godefroid et al. (2015)
*Cameraria ohridella*	152	0.97	Barredo et al. (2015)
*Ceratitis fasciventris*	49	0.82 *	Godefroid et al. (2015)
*Comperiella calauanica*	20	0.966	Current study
*Hylobius abietis*	677	0.93	Barredo et al. (2015)
*Lobesia botrana*	95	0.970 *	Lv et al. (2012)
*Phenacoccus solenopsis*	111	0.895	Fand et al. (2014)
*P. solenopsis*	201	0.92	Wei et al. (2017)
*Polistes dominula*	9246	0.982 *	Howse et al. (2020)
*Ricania shantungensis*	43	0.79	Baek et al. (2019)

* Not indicated if reported AUC value refers to test AUC or training AUC.

## Data Availability

The data presented in this study are available on request from the corresponding authors.
